# Herbal medicine use and renal dysfunction among persons living with HIV on Tenofovir-based ART in Kampala, Uganda

**DOI:** 10.1371/journal.pone.0343124

**Published:** 2026-02-18

**Authors:** Stuart Niwagaba, Anthony Muyunga, Douglas Bulafu, Joslyline Lydia Nakanwagi, Sandra Lunkuse, Faizo Kiberu, Shivan Nuwasiima, Elizabeth Nagawa, Irene Karungi, Vella Ayugi, Patience Muwanguzi, Henry Kyeyune, Caroline Birungi

**Affiliations:** 1 Department of Biochemistry, School of Biosciences, College of Natural Sciences, Makerere University, Kampala, Uganda; 2 Infectious Diseases Research Collaboration, Kampala, Uganda; 3 Clinical Epidemiology Unit, Department of Internal Medicine, School of Medicine, College of Health Sciences, Makerere University, Kampala, Uganda; 4 Reach Out Mbuya Community Health Initiative, Kampala, Uganda; 5 Department of Disease Control and Environmental Health, School of Public Health, College of Health Sciences, Makerere University, Kampala, Uganda; 6 MRC/UVRI and LSHTM Uganda Research Unit, Entebbe, Uganda; 7 Imperial Centre for cardiovascular disease prevention, Department of Primary care and Public Health, School of Public Health, Imperial College London, London, United Kingdom; 8 Department of Nursing, School of Health Sciences, College of Health Sciences, Makerere University, Kampala, Uganda; 9 Department of Pharmacy, School of Medicine, College of Health Sciences, Makerere University, Kampala, Uganda; 10 Department of Psychiatry, School of Medicine, College of Health Sciences, Makerere University, Kampala, Uganda; 11 Mulago Immune Suppression Syndrome Clinic, Mulago National Referral Hospital, Kampala, Uganda; Debre Markos University, ETHIOPIA

## Abstract

**Introduction:**

HIV/AIDS remains a major public health issue, with about 1.4 million people infected in Uganda by 2020. In rural western Uganda, 57.6% of people living with HIV (PLWH) use herbal medicine (HM) alongside antiretroviral therapy (ART). Combining HM with a tenofovir disoproxil fumarate (TDF)-based ART regimen may increase risks of renal dysfunction, death, and treatment costs, highlighting the need to study their combined effects.

**Objective:**

To determine the prevalence of herbal medicine use and its association with renal dysfunction among patients on tenofovir antiretroviral treatment-based regimen at ISS clinic Mulago.

**Methods:**

A cross-sectional study at MJAP ISS-clinic involved 414 HIV/AIDS patients on TDF-based ART from March–May 2023. Data on herbal medicine use and ART regimen were collected via interviews; blood samples were taken for renal function. Additional clinical and demographic data were extracted from records. All data were entered into Epidata and analyzed using STATA version 17.

**Results:**

We enrolled 414 participants with median age (interquartile range) of 36 (30,44) years, majority were female 290 (70.1) and were on firstline regimen 392 (94.7). The prevalence of herbal medicine use was 70.8% (95% CI 66.2–74.9). The commonly used herbal medicines reported were concoction/crude, medicated clay and powdered products. The overall renal dysfunction prevalence was 22.5% (95 CI 18.7–26.8) The median serum creatinine levels among herbal medicine users was 96 µmol/litre and 88.8 µmol/litre among non-users. There was a significant difference in the median serum creatinine in the two groups (P = 0.028). There was no significant difference in the urea levels in the two groups (2.99 mmol/litre in herbal medicine users versus 2.84 mmol/litre in non-users, P = 0.689). Herbal medicine use was significantly associated with renal dysfunction (aPR-2.31, 95% CI 1.35–3.97). Other factors that were associated with renal dysfunction were age (aPR-1.54, 95% CI 1.08–2.22) sex (aPR-0.52, 95% CI 0.33–0.83), hypertension (aPR-3.43, 95% CI 2.47–4.76) and diabetes (aPR-1.79, 95% CI 1.39–2.31).

**Conclusion:**

Health care workers should screen for herbal medicine use among ART patients and regularly monitor their renal function to detect dysfunction. The Ministry of Health and health care workers should also emphasize educating patients about the potential risks of combining herbal medicine with ART.

## Introduction

HIV/AIDS remains a public health concern on a global scale. As of 2021, approximately 38.4 million individuals were living with HIV, and around 680,000 people lost their lives due to AIDS-related illnesses [1]. Sub-Saharan Africa bears the heaviest burden accounting for more than two-thirds of all individuals living with HIV [2]. In Uganda, an estimated 1.4 million people are infected with HIV, indicating a persistent and substantial national burden [3].

The mainstay of HIV treatment is antiretroviral therapy (ART), with the current first-line regimens predominantly based on dolutegravir (DTG) [4]. While these combinations, particularly those containing tenofovir disoproxil fumarate (TDF), have shown high efficacy in viral suppression, long-term use has been associated with renal toxicity and other adverse effects [5]. Consequently, managing the side effects related to ART presents an ongoing challenge for healthcare providers and patients alike, leading many people living with HIV (PLWH) to seek complementary solutions, including herbal remedies [6].

Herbal medicine is commonly turned to by patients seeking to alleviate ART-induced side effects, manage co-existing conditions like hypertension, or generally improve their immunity [7]. According to the World Health Organization (WHO), herbal medicine includes herbs, herbal materials, preparations, and finished products that contain parts or materials of plants used for medicinal purposes worldwide [8].

The global use of herbal medicine among PLWH is notably widespread, with prevalence rates ranging from 50% to as high as 95% in some regions [9]. In Africa, about 80% of individuals utilize herbal remedies, largely due to their affordability, accessibility, and cultural acceptance [10]. In Uganda specifically, the prevalence of herbal medicine use among individuals on ART stands at 57.6%. Factors influencing this use include age, marital status, duration on ART, perceived side effects, educational background, occupation, and individual knowledge and expectations regarding herbal products [11].

Despite its common use, herbal self-medication is often underestimated by healthcare providers because patients rarely disclose such practices [12], This lack of communication is concerning, given the potential for adverse effects and toxicity. Herbal remedies may interact negatively with ART medications, decreasing their serum concentrations and possibly leading to treatment failure and further complications due to toxic substances [13]. Many herbal extracts contain a mix of phytochemicals, such as saponins, tannins, and steroids, which may disrupt drug metabolism [14], For instance, St. John’s wort has been shown to drastically reduce levels of the ART drug indinavir through interactions mediated by hyperforin [15]. Moreover, herbal medicine has been identified as a risk factor for both hepatic and renal dysfunction in humans [7].

TDF, though widely used as a first-line nucleoside reverse transcriptase inhibitor (NRTI), is linked to nephrotoxicity in between 0.5% and 45% of patients, despite its perceived safety [16]. It has also been reported to slightly but significantly lower creatinine clearance in PLWH, reflecting early signs of kidney dysfunction [17].

While herbal medicines are often viewed as safe, their misuse or high dosages can lead to health risks due to the presence of toxic compounds in certain extracts [12]. Many of these plant-based remedies can induce or inhibit cytochrome P450 enzymes, affecting drug metabolism through herb-drug interactions [18]. Though TDF is widely used as a safe first-line NRTI, nephrotoxicity occurs in 0.5–45% of HIV-positive patients [16].

Currently, there is limited information regarding the prevalence of herbal medicine use and its relationship with renal dysfunction among patients on TDF-based ART regimens. This knowledge gap may impede optimal patient care in health settings.

Given that herbal and ART drugs may exert additive renal stress, their concurrent use could heighten the risk of renal impairment, contribute to treatment failure, elevate mortality rates, and increase healthcare costs. However, this potential interaction remains poorly investigated in clinical settings.

The popularity of herbal medicine continues to rise among PLWH, approximately 80% of patients self-medicate, with up to 50–80% not informing healthcare providers of their herbal use [19]. Many believe herbal treatments are safe, natural, and culturally suitable for treating illness [20]. Unfortunately, the absence of national guidelines regulating herbal medicine use among HIV-positive individuals contributes to its widespread, and sometimes unmonitored, use. The findings are intended to inform policy formulation, help establish clear management guidelines, and guide healthcare workers in offering patient education and routine renal function monitoring to improve outcomes for patients using both ART and herbal medicines. This study aimed to determine the prevalence of herbal medicine use and its possible association with renal dysfunction among PLWH on TDF-based ART.

## Methods

### Study design

A cross-sectional study was conducted with both descriptive and analytical components employing quantitative methods of data collection.

### Study setting

The study was conducted at Makerere University Joint AIDS Program (MJAP) ISS Clinic Mulago among people receiving routine ART care services between May 2023 and July 2023.

The MJAP ISS clinic is the largest HIV clinic in Uganda serving about 20000 PLWHIV with about 16,500 persons on TDF ART regimen. ISS Clinic Mulago is a private not for profit Non-Governmental Organization that provides free comprehensive HIV/AIDS services in Mulago Hospital. The clinic is located along Upper Mulago hill road within Kawempe South division in Kampala District.

HIV/AIDS Clinical services are provided to patients daily with exceptions of weekend and public holidays. Services offered include, HIV testing, counselling, end mother to child transmission services, gender-based violence services, integrated TB/HIV, integrated HIV/Hypertension, laboratory testing, ART drug refills, key populations and priority population services, community facility models and HPV screening, among others.

The clinic was chosen as it is centrally located and serves persons from different economic and social life styles within the hospital and surrounding communities. The facility offers a lot of free services hence accessible for all and majority of its clients are on TDF based regimen ART.

### Study population

All people above 18 years living with HIV/AIDS on TDF based ART receiving routine care at MJAP ISS clinic between May 2023 and July 2023 who fulfilled the eligibility criteria of the study.

### Eligibility criteria

All people above 18 years living with HIV/AIDS on TDF based ART receiving routine care at MJAP ISS clinic between May 2023 and July 2023 and that provided written informed consent to participate in the study.

Patients who were ill and unable to withstand and respond in to the questionnaire interview process.

### Sample size determination and sampling procedure

Sample size was estimated using Kish Leslie’s formula [21]. Considering Prevalence value of 57.6% from a study conducted by Ayesiga and colleagues [11]. This resulted in the sample size of 414 participants.

A systematic random sampling technique was used to select 414 study participants. Every fifth participant was selected from a sampling frame based on patient orders when coming to the clinic for follow-up appointments. The first participant was randomly selected from the first three study participants using simple random sampling by writing numbers 1–5 on pieces of paper and randomly selecting one and then data was collected and continued for every fifth study participant. In case the fifth was not eligible, the next participant was considered.

### Study variables

We collected data on socio-demographics including Age, gender, weight and highest level of education, clinical factors comprised of Comorbidities, ART regimen, herbal medicine use, duration on ART regimen and duration using herbal medicine. We also gathered information potential confounding variables on alcohol consumption, smoking, hypertension, diabetes mellitus, tuberculosis and drugs that increase the risk of kidney dysfunction such as Non- steroidal anti-inflammatory drugs (NSAIDS), aminoglycosides and illicit drugs. Possible confounders like viral load, CD4 count, and concomitant drugs were not assessed in this study.

The outcome variable was herbal medicine use and in this study it was operationally defined as the oral use of crude or finished products whose active ingredients are of plant origin such as leaves, roots, stems, flowers and seeds. It was assessed and measured using a questionnaire as someone on TDF based regimen who had been using herbal medicine in the last 6 months irrespective of the purpose of usage. Herbal medicine was defined as any concoction/crude, medicated clay and powdered products that contain active ingredient of plant origin. Herbal medicines were categorized into three forms based on participant report crude/concoction (fresh or boiled plant materials), medicated clay preparations, and powdered plant products.

Also renal function parameters that were measured and compared included serum creatinine and urea. Estimated glomerular filtration rate was computed using the CKD-EPI creatinine equation 2021 in milligrams per deciliters (mg/dL) and Age of subject in years to assess renal dysfunction status as either Yes (if eGFR was below 60 mL/min/1.73 m^2^ or No (if eGFR was 60 mL/min/1.73 m^2^ and Above [22].

### Data collection

Participants were given a unique identification number just after consenting to participate in the study before the interview. Data was collected by the principal investigator (PI) and three registered nurses. The questionnaires were pretested on ten eligible participants to assess their feasibility and consistency. The recruitment period of the study started on 22/05/2023 and ended on 14/07/2023.

Four milliliters of blood samples were collected from the non-dominant arm or on patient’s preference by the research nurses into a plain vacutainer (Red top), well labelled with participant number and transported to the laboratory in a cool box. Laboratory renal function parameter measurements were done by a qualified laboratory technologist using blood serum on a well calibrated COBAS integra 6000 Chemistry Analyzer machine. Participant’s results were recorded in a laboratory results sheet section attached to the Questionnaire. The creatinine level value in mg/dL and age in years were used to compute the eGFR using the CKD-EPI formula.

### Data management

All research nurses were trained and required to sign on the confidentiality form from the principal investigator. All raw data was stored securely on a password protected laptops, an external hard drive and it was only be accessible to the study team.

Double data entry was performed using Epidata version 4.6. The data was cleaned and then exported to STATA version 17.0 statistical software for analysis.

### Data analysis

Data was analyzed using STATA version 17.0. Univariate analysis was done to summarize continuous variables using means, standard deviations, medians, and ranges. Frequencies, proportions, and percentages were used to summarize any categorical variables.

The prevalence of herbal medicine use among patients on TDF-based ART regimen was reported as a percentage with the numerator being the total number of people assessed to be herbal medicine users and denominator being the total number of all study participants. Prevalence was reported for the different study sub-populations and summarized.

The Wilcoxon ranksum test was done to compare if serum Creatinine and Urea were statistically different between TDF based regimen Herbal medicine users and Non-users at ISS-clinic Mulago as the normality assumption of the variables between the two groups were violated.

To determine if there is an association between herbal medicine use and renal dysfunction, modified Poisson regression analysis with robust standard errors was used since renal dysfunction was categorized into a binary outcome and herbal medicine use was considered as the main predictor variable.

Assumptions for suitability of modified Poisson regression were assessed and fulfilled.

Bivariate analysis was performed for each of the independent variables to determine which ones were to be included in the multivariate analysis. Variables with p-value < 0.2 were included in the multivariate analysis. At multivariate analysis, variables with a p-value <0.2 were used form interaction terms with the main predictor variable herbal medicine use form a full model. A chunk test was done to assess for interaction.

Confounding was assessed as a difference >10% between the crude and adjusted measure of association in the models.

### Quality control

Pretesting of the standard semi – structured questionnaires was conducted by the PI on a sample of 10 participants to evaluate the appropriateness of the variables to be collected. Research nurses received a two-days training about the questionnaire and tools to be used in the study. Double entry of the collected data was done in order to minimize errors. The structured questionnaires and consent forms were translated to Luganda, the local language. The filled questionnaires were assessed daily for completeness by the principal investigator. All study materials were stored under lock and key by the principal investigator.

A unique code was assigned to the participants for identification after consent. Double entry method for the data was done to ensure completeness and correctness daily. An independent research colleague was used to recheck the data prior to analysis.

### Ethical consideration

Ethical approval to conduct the study was sought from the Clinical Epidemiology Unit, MJAP ISS Clinic administration and School of Medicine Research and Ethics Committee (SOMREC) with SOMREC-2023–570 number. Informed written consent was obtained from all study participants.

Participants who were found with out of range renal function parameter values were referred to the clinicians for management.

## Results

### Socio-demographic, clinical and herbal medicine use characteristics of participants

The study participants had a median (IQR) age of 36 (30,44) years and median weight of 66 (59,75) kilograms. Majority of the study participants were females (n = 290, 70.1%) and had attained secondary education (n = 154, 37.2%).

Majority of the study participants (n = 392, 94.7) were on a first line regimen and had a median duration of 3 (2,5) years on ART. Majority of the study participants were non-alcohol users (n = 300, 72.5%) and 9.2% (n = 38), had hypertension.

Concoction (243, 82.9%) was the most used herbal medicine, most of the study participants (n = 274, 93.5) had not disclosed to their clinicians that they used herbal medicine and only 29.6% (n = 81) were willing to disclose their herbal use status indicated in Table 1.

**Table 1:** Socio-demographic and clinical characteristics of study participants, N = 414.

Variable	Category	Median (Q1, Q3)	Overall n (%)
Age		36 (30,44)	
Sex	Female		290 (70.1)
	Male		124 (29.9)
Weight		66 (59,75)	
Education level	No Education		34 (8.2)
	Primary		151 (36.5)
	Secondary		154 (37.2)
	Tertiary		75 (18.1)
ART Regimen	First line		392 (94.7)
	Second line		22 (5.3)
Duration on ART		3 (2,5)	
Hypertension	Yes		38 (9.2)
	No		376 (90.8)
Diabetes	Yes		17 (4.1)
	No		397 (95.9)
Alcohol use	Yes		114 (27.5)
	No		300 (72.5)
Herbal Product	Concoction/Crude		243 (82.9)
	Medicated clay		35 (11.9)
	Powdered		15 (5.1)
Clinician Awareness	No		274 (93.5)
	Yes		19 (6.5)
Intention to declare	No		193 (70.4)
	Yes		81 (29.6)

### Prevalence of herbal medicine use

The overall prevalence of herbal medicine use was 70.8% (95% CI 66.2–74.9). The prevalence was high among females (72.8%, 95% CI 67.3–77.6) and to those individuals aged above 36 years (77.1%, 95% CI 70.8–82.4). The prevalence of herbal medicine use was also high among patients on second line ART (77.3%, 95% CI 55.6–90.2) as indicated in Table 2.

**Table 2:** Prevalence of herbal medicine use of study participants, N = 414.

Variable	Category	N	Herbal medicine users, n (%)	95% CI
Overall Prevalence^#^		414	**293 (70.8)**	66.2–74.9
Age	36 and Below	213	138 (64.8)	58.1–70.9
	Above 36	201	**155 (77.1)**	70.8–82.4
Sex	Female	290	**211 (72.8)**	67.3–77.6
	Male	124	82 (66.1)	57.4–73.9
Education level	No Education	34	15 (44.1)	28.6–60.9
	Primary	151	**114 (75.5)**	67.9–81.7
	Secondary	154	113 (73.4)	65.8–79.8
	Tertiary	75	51 (68.0)	56.6–77.6
ART Regimen	First line	392	276 (70.4)	65.7–74.7
	Second line	22	**17 (77.3)**	55.6–90.2
Hypertension	No	376	263 (70.0)	65.1–74.4
	Yes	38	**30 (79.0)**	63.2–89.1
Diabetes	No	397	278 (70.0)	65.3–74.3
	Yes	17	**15 (88.2)**	63.1–97.1
Alcohol use	Yes	114	**90 (78.9)**	70.5–85.5
	No	300	203 (67.7)	62.2–72.7

#Overall, prevalence of of herbal medicine use among the 414 participants.

### Median serum renal function parameters of TDF-based art regimen herbal medicine users and non-users at ISS-clinic mulago

The overall prevalence of renal dysfunction among 414 participants was 22.5% (95 CI 18.7–26.8). There was a significant difference in the median serum levels of creatinine between herbal medicine users and non-users. However, there was no significant difference in the median serum urea levels as shown in (Table 3).

**Table 3:** Comparison of the median serum renal function parameters between Herbal medicine users and Non-users, n = 414.

Renal function parameters	Herbal medicine users	Non-herbal medicine users	P-value
Median serum Creatinine (µmol/L)	96	88.8	**0.028**
Median serum Urea (mmol/L)	2.99	2.84	0.689

### Association between herbal medicine use and renal dysfunction among patients on tdf-based art regimen at ISS-clinic mulago

At bivariate analysis, herbal medicine use (PR-2.79 95% CI 1.58–4.92), age (PR-1.93 95% CI 1.32–2.81), sex (PR-0.52 95% CI 0.32–0.85), secondary education level (cPR0.54 95% CI 0.32–0.92) and tertiary education level (0.45 95%CI 0.23–0.87), ART regimen (PR-1.91 95% CI 0.94–2.63), hypertension (PR-3.84 95% CI 2.83–5.22), diabetes (PR-1.58 95% CI 1.24–2.02) and herbal product were significantly associated with renal dysfunction. Furthermore, at multivariate analysis herbal medicine use (aPR-2.31 95% CI 1.35–3.97), age (aPR-1.54 95% CI 1.08–2.22), sex (aPR-0.52 95% CI 0.33–0.83), hypertension (aPR-3.43 95% CI 2.47–4.76) and diabetes (aPR-1.79 95% CI 1.39–2.31) were significantly associated with renal dysfunction (Table 4).

**Table 4:** Factors associated with renal dysfunction among 414 PLWH on TDF based ART regimen.

Variable	Category	Renal dysfunction		cPR	95% CI	P-value	aPR	95% CI	P-value
		Yes	No						
Herbal Medicine use	No			Ref					
	Yes			2.79	1.58–4.92		2.31	1.35–3.97	0.002
Age	36 and Below	33 (15.5)	180 (84.5)	Ref			Ref		
	36 Above	60 (29.9)	141 (70.1)	1.93	1.32–2.81	**0.01**	1.54	1.08–2.22	0.018
Sex	Female	76 (26.2)	214 (73.8)	Ref			Ref		
	Male	17 (13.7)	107 (86.3)	0.52	0.32–0.85	**0.01**	0.52	0.33–0.83	0.006
Weight	66 Below	46 (20.9)	174 (79.1)	Ref					
	Above 66	47 (24.2)	147 (75.8)	1.16	0.81–1.66	0.42			
Education level	No Education	13 (38.2)	21 (61.8)	Ref					
	Primary	35 (23.2)	116 (76.8)	0.61	0.36–1.02	**0.06**			
	Secondary	32 (20.8)	122 (79.2)	0.54	0.32–0.92	**0.02**			
	Tertiary	13 (17.3)	62 (82.7)	0.45	0.23–0.87	**0.02**			
HM Duration	Less than 2 years	53 (27.0)	143 (73)	Ref					
	More than 2 years	28 (28.9)	69 (71.1)	1.07	0.72–1.57	0.74			
ART Regimen	First line	84 (21.4)	308 (78.6)	Ref					
	Second line	9 (40.9)	13 (59.1)	1.91	1.11–3.27	**0.02**			
Alcohol use	No	65 (21.7)	235 (78.3)	Ref					
	Yes	28 (24.6)	86 (75.4)	1.13	0.76–1.67	0.53			
Hypertension	No	67 (17.8)	309 (82.2)	Ref			Ref		
	Yes	26 (68.4)	12 (31.6)	3.84	2.83–5.22	**<0.01**	3.43	2.47–4.76	<0.001
Diabetes	No	84 (21.2)	313 (78.8)	Ref			Ref		
	Yes	9 (52.9)	8 (47.1)	1.58	1.24–2.02	**<0.01**	1.79	1.39–2.31	<0.001
herbal Product	Concoction/Crude	66 (27.2)	177 (72.8)	Ref					
	Medicated clay	14 (40.0)	21 (60.0)	1.47	0.93–2.32	**0.09**			
	Powdered	1 (6.7)	14 (93.3)	0.25	0.04–1.65	**0.15**			

## Discussion

The prevalence of herbal medicine use in this cross-sectional study was found to be 70.8% (95% CI 66.2–74.9). This means that about 7 in every 10 people living with HIV on TDF based ART regimen use herbal medicine. These findings are higher than 57.6% reported by Ayesiga [11] and this could be because our study was conducted in an urban setting where there are a lot of herbal products readily available, improved branding and packaging that are being advertised across social media, televisions, radios and even in print media compared to that done in a rural setting in Kabarole.

Secondly, Our study findings are higher than those reported from a descriptive, cross-sectional study done in two geographical areas in the United States of America among men with HIV infection that found the prevalence of herbal medicine use 69%, (n = 301) [23]. This study was done among men only and the case would be different if done in a mixed population.

The prevalence reported by our study could be an underestimate because of self-reporting as there could be some fears to disclose as evidenced by the approximately 70% who had no intention to disclose as shown in Table 1. However, the findings are considered true for those participants that reported to be herbal medicine users as they had to respond to other questions for more details such as how often, duration of use and type of herbal product being used.

Contrary, a cross sectional study conducted in urban areas of Enugu Nigeria among all people (Both HIV positive and negative) that reported a prevalence of herbal medicine at 84% [24]. This was because this study was conducted in the general population hence not specific for people living with HIV on TDF-based ART just like our study.

These study findings imply that PLHIV do not adhere properly to the instructions given to them by clinicians during the health education talk sessions at ART clinics. More emphasis and time can be put to discourage the use of herbal medicine in combination with ART because of the likely negative associated outcomes such as renal dysfunction. Therefore, Ministry of health (MOH) should develop educational campaigns to provide information and awareness on the potential risks and limitations of using herbal medicine in combination with ART such as the herbal medicine-ART drug interactions which should not be under looked.MOH should establish ways in the HIV care and management policy on how to monitor adverse events related to the use of herbal medicine among PLHIV.

### Comparison of serum creatinine and urea between herbal medicine users and non-herbal medicine users

The findings from this cross-sectional study show that there is a significant difference in the median serum creatinine and no significant difference in urea levels of herbal medicine users when compared to the non-herbal medicine users. Elevated serum creatinine reflects reduced glomerular filtration rate due to impaired renal function. It accumulates when glomerular, tubular, or perfusion-related kidney damage limits creatinine clearance. Thus, increased creatinine serves as a robust biomarker of the presence and progression of renal dysfunction [25].

The findings imply that individuals who use herbal medicine in combination with ART are likely to have significantly elevated serum creatinine levels compared to non-herbal medicine users on ART. Further still, individuals who use herbal medicine in combination with ART are likely to have a non-significantly elevated serum urea levels compared to non-herbal medicine users on ART.

The possible causes for the differences in the above findings could be due to different Pharmacokinetic interactions as some herbs could potentially alter the clearance or renal excretion of creatinine, leading to variations in creatinine levels [26].

Additionally, the differences in levels could be due to variations in the quality, potency, and composition of herbal medicines used by different individuals. Different herbal preparations might contain varying amounts of active compounds, potentially resulting in diverse effects on renal function. Variability in the specific herbs used, preparation methods, dosage, and duration of use could contribute to the observed differences.

The findings are similar to a study conducted to assess the effects of aqueous extract of *Olea europaea* leaves on the liver and kidney function parameters that reported a significant increase in the serum urea and creatinine in swiss albino mice [27]. Although the absolute difference in median serum creatinine values between herbal medicine users and non-users was modest, this difference is clinically meaningful because even small elevations in creatinine among individuals on tenofovir-based regimens may signal early nephron injury. This is supported by the significantly higher prevalence of renal dysfunction (eGFR < 60 mL/min/1.73m²) among herbal medicine users. Therefore, the observed biochemical difference should be interpreted in the context of cumulative renal risk rather than as an isolated laboratory value [28].

### Association between herbal medicine use and renal dysfunction among patients on tdf-based art regimen

#### Herbal medicine use.

The findings from our study show that herbal medicine use in combination with TDF ART is associated with renal dysfunction. The results show that herbal medicine users on ART are 2.31 times more likely to have renal dysfunction compared to the non-herbal medicine users.

This could be because combinational use of herbal medicine and ART may lead to accumulation of toxic waste substances from the body metabolism that damage the kidney nephrons and lower its efficiency resulting into renal dysfunction. Additionally, some plant extracts have been reported to contain nephrotoxic compounds that cause damage to cells such as aristolochic acids by [29].

Secondly, majority of our study participants reported to be using non-defined (Non-standardized) doses of crude plant concoctions and medicated clay in large doses more especially among women and these cumulatively increase the risk of renal dysfunction due to the additional stress on the kidneys as they filter and excrete the wastes.

The findings are similar to those from a study conducted using longitudinal health insurance database in Taiwan that had 24971 participants and reported association between a prescribed Chinese herbal medicine and renal disease [30].

Additionally, our study findings show that the use medicated clay increases the likelihood of renal dysfunction compared to crude herbs and the use powdered herbal products show they reduce the likelihood of renal dysfunction compared to crude herbs. This indicates that different herbal forms have different likelihood outcomes of renal dysfunction. This shows that not all herbal products may be bad for use and therefore they should not be considered as one construct of herbal medicine but instead be tested independently.

### Limitations of the study

The study may be subject to information bias, as self-reported herbal medicine use could be underreported due to social desirability and participants’ difficulty accurately identifying specific preparations, potentially leading to an underestimation of prevalence and misclassification of exposureThere could have been some confounding as not all variables were assessed and analyzed and these included other treatment medication, viral load status, CD4, diet among others.

The study was not able to assess alcohol use using a standard tool to do quantification as it focused on knowing if an individual used alcohol or not.

Temporal relationship could not be ascertained to clearly understand if renal dysfunction preceded herbal medicine use or vise-versa as well as causal relationship.

There could have been a possibility of under diagnosis of renal dysfunction as we defined renal dysfunction based on eGFR and did not look at proteinuria.

HIV drugs are usually in combinations and different combinations may affect renal dysfunction in different ways.

The results of the study can be generalizable to urban-based public facilities in Uganda which may not be the case in other parts of the country.

However, above limitations and biases highlighted did not affect the study substantially hence the findings are valid.

## Conclusion and recommendations

The study found that seven in every 10 PLWH on a TDF-based ART regimen use herbal medicine. Herbal medicine users had significantly different median serum creatinine levels compared to non-users, but no significant difference in median serum urea levels. The use of herbal medicine alongside ART was significantly linked to renal dysfunction, which was also associated with factors such as age, sex, hypertension, and diabetes mellitus.

Healthcare workers should educate patients on the risks of using herbal medicine alongside ART, while clinicians should routinely assess renal function in these patients to improve detection of renal dysfunction. The Ministry of Health and the National Drug Authority need to implement strategies to monitor adverse events from herbal medicine use among PLWH. Additionally, MOH should provide guidelines for producers of these products to undergo some form of training or standardization for better and safer products.

Further follow-up studies are required to account for factors linked to renal dysfunction and to clarify the temporal relationship between herbal medicine use and renal outcomes.

Additionally, pharmacological research is essential to explore the risks, benefits, and interactions whether present or absent between commonly used herbal medicines and ART in PLHIV.

## Supporting information

S1 File STROBE checklist. (DOC)

S2 File Supplementary file (Questionnaire). (PDF)

S3 File Dataset file. (XLSX)
