# Supplementary material for: Herbal medicine use and renal dysfunction among persons living with HIV on Tenofovir-based ART in Kampala, Uganda
Source: PLoS One. 2026 Feb 18;21(2):e0343124. doi: 10.1371/journal.pone.0343124 (PMC12915923; doi:10.1371/journal.pone.0343124)
Supplement: S2 File — (PDF) [file pone.0343124.s002.pdf]

APPENDICES

Appendix A: Eligibility Criteria assessment form

This form aids in the selection of participants eligible for the enrollment into the study.

To be enrolled, participants should have fulfilled the eligibility criteria.

Eligibility assessment table

Participant unique code		
**Inclusion criteria**	**Yes**	**No**
Participant on TDF-based regimen.		
Has given informed consent.		
**Exclusion criteria**	**Yes**	**No**
Patients known to be on renal dysfunction (AKD or CKD) treatment.		
Patients with history of renal dysfunction.		
Patients unable to adhere to study procedures.		

Eligible if participant meets all the inclusion criteria

Eligibility status

Eligible ☐ Not Eligible ☐

Study staff initials………………

Signature……………………………………Date…………………………………

Appendix B: Study Questionnaire

Study title: Prevalence of herbal medicine use and its association with renal dysfunction among patients on tenofovir antiretroviral treatment-based regimen at ISS clinic Mulago.

1.	**Unique participant code**	
	**Data collector information**	
2.	Study staff initial	
3.	Date of collection	
	**Section A: Demographic characteristics**	
4.	Age in completed years	
5.	Sex	• Male<br>• Female
6.	Weight in Kgs	
7.	Level of education	• No formal education<br>• Primary<br>• Secondary<br>• Tertiary
	**Section B: Clinical characteristics**	
8.	Herbal medicine use	• Yes<br>• No
9.	what is the duration while using Herbal medicine if yes?	
**10.**	Who advised/prescribed the use herbal medicine?	• Friends/relatives<br>• Spouse<br>• Clinician<br>• Herbalist
**11.**	How often do/did you use the herbal medicine?	• Once a day<br>• 2-3 times a day<br>• Any time you feel like
**12.**	Current ART Regimen	
**13**	Duration on current ART regimen?	
14.	Underlying comorbidities	• Hypertension<br>• Diabetes Mellitus<br>• Tuberculosis<br>• Cardiovascular diseases<br>• Other……………(specify)
15.	Consumption of alcohol	• Yes<br>• No
16.	Smoking	

17.	Herbal medicine name	
18.	Is your clinician aware that you are using herbal medicine in combination with ART?	
19.	Do you intend to inform your clinicians about it?	

APPENDIX C: Study questionnaire in Luganda

Okunonyereza ku bungi bwabantu abakozesa eddagala lye'kinaasi nokumanya oba waliwo akakwatte wakati wokukozesa eddagala lino nokwononeka kwe nsigo mubantu abali ku ddagala lyakawuka ka mukenenya elyekika kya tenofovir ku ISS clinic Mulago

1.	**Koodi yo'mulwadde**	
	**Ebikwata ku akunganya**	
2.	Initial zo'mukunganya	
3.	Enaku zo'mwezi	
	**Akatundu A: Ebikwata ku mulwadde**	
4.	Emyaka gyo mulwadde	
5.	Obutonde	• Musajja<br>• Mukazi
6.	Obuzito mu Kgs	
7.	Okusoma kwo	• Teyasoma<br>• Pulayimale<br>• Sekendule<br>• Yunivasite
	**Akatundu B: Obujanjabi bwo mulwadde**	
8.	Okozesa eddagala lye'kinansi	• Yee<br>• Nedda
9.	Eddagala lye'kinansi olikozeseza kumala banga ki	
**10.**	Ani yalikulagirila	• Abemikwano ne nganda<br>• Omwagalwawo<br>• Omusawo we'kizungu<br>• Omusawo we'kinansi
**11.**	Eddagala lyekinansi olikoseza emirundi emeeka	• Omurundi guumu mulunaku<br>• Wakati wemirundi ebiri kwe'satu<br>• Wenjagalidde okulikozesa
**12.**	Eddagala lyakawuka kamukenenya lyokozesa kati	•
**13.**	Olikozeseza kumala banga ki	•
14.	Endwadde endala zolina	• Pulesa<br>• Sukali<br>• Akafuba<br>• Endwadde zo mutima

		• Endala yona…………..
15.	Okozesako kumwenge	• Yee<br>• Nedda
16.	Okozesako ku sigala	
17.	Elinya lyeddagala lyo’kozesa	
18.	Abasawo abakujanjaba bakimantiko nti okozesa eddagala lino awamu nezungu	
19.	Osubira okugamba abasawo nti okozesako ku ddagala lino awamu nezungu	
